# Ultrafest: A Novel Approach to Ultrasound in Medical Education Leads to Improvement in Written and Clinical Examinations

**DOI:** 10.5811/westjem.2014.11.23746

**Published:** 2014-12-15

**Authors:** Kiah Connolly, Lancelot Beier, Mark I. Langdorf, Craig L. Anderson, John C. Fox

**Affiliations:** University of California, Irvine, School of Medicine, Irvine, California

## Abstract

**Introduction:**

Our objective was to evaluate the effectiveness of hands-on training at a bedside ultrasound (US) symposium (“Ultrafest”) to improve both clinical knowledge and image acquisition skills of medical students. Primary outcome measure was improvement in multiple choice questions on pulmonary or Focused Assessment with Sonography in Trauma (FAST) US knowledge. Secondary outcome was improvement in image acquisition for either pulmonary or FAST.

**Methods:**

Prospective cohort study of 48 volunteers at “Ultrafest,” a free symposium where students received five contact training hours. Students were evaluated before and after training for proficiency in either pulmonary US or FAST. Proficiency was assessed by clinical knowledge through written multiple-choice exam, and clinical skills through accuracy of image acquisition. We used paired sample t-tests with students as their own controls.

**Results:**

Pulmonary knowledge scores increased by a mean of 10.1 points (95% CI [8.9–11.3], p<0.00005), from 8.4 to a posttest average of 18.5/21 possible points. The FAST knowledge scores increased by a mean of 7.5 points (95% CI [6.3–8.7] p<0.00005), from 8.1 to a posttest average of 15.6/21. We analyzed clinical skills data on 32 students. The mean score was 1.7 pretest and 4.7 posttest of 12 possible points. Mean improvement was 3.0 points (p<0.00005) overall, 3.3 (p=0.0001) for FAST, and 2.6 (p=0.003) for the pulmonary US exam.

**Conclusion:**

This study suggests that a symposium on US can improve clinical knowledge, but is limited in achieving image acquisition for pulmonary and FAST US assessments. US training external to official medical school curriculum may augment students’ education.

## INTRODUCTION

Physician-performed bedside ultrasound (US) for diagnosis and procedural guidance is valuable, with studies from many specialties showing improved evaluation of patient pathology.^1^,^2^ US is portable, relatively inexpensive, and has no radiation or health risks. Furthermore, technology advances and declining machine costs have improved bedside applicability.^3^ The major limitation to physician-performed US in standard practice is proficiency at image acquisition and interpretation. Integration of US training into medical education is a next critical step. While some medical students already receive limited or even advanced US training as part of their curriculum, most do not. This study evaluated the effectiveness of hands-on training at a bedside US symposium (“Ultrafest”) in improving clinical knowledge and image acquisition skills of medical students.

## METHODS

“Ultrafest,” created in May 2012, was the nation’s first multidisciplinary bedside US symposium directed to medical students who lacked formal curricula in their native schools. Attendance reached over 200 student attendees in 2012, so the conference was repeated in February 2013 where we studied the educational impact of the symposium reported here.

The conference was free and accepted students from allopathic, osteopathic and physician assistant programs. The symposium was tailored to the interests of each student, allowing them to choose five of 12 workshops to most effectively build skills and meet their peak interests. All students were instructed to watch subject-specific online tutorials created by the US director prior to arrival to maximize hands-on experience at the event. Workshops offered were cardiology, anesthesia, pulmonary, male genitourinary, female pelvis, question-and-answer image review, pediatrics, obstetrics, musculoskeletal, trauma simulation, hepatobiliary and vascular. Workshops featured 32 live models including multiple live pelvic, pregnant, and male genitourinary models, as well as musculoskeletal and hepatobiliary pathology. Thirty-six US machines were used in addition to multiple “Sonosim” ultrasound simulators to depict real-time trauma and cardiac pathology. Phantom task trainer models were used to enable procedure practice (central line placement and thoracentesis), and viewing of pathology in transvaginal, Focused Assessment with Sonography in Trauma (FAST), and pleural effusion.^4^

More specifically the technology utilized included Sonosim “Editions” (Santa Monica, CA), Blue Phantom Combination IUP Ectopic Pregnancy Transvaginal Model (item #BPOB1227), Blue Phantom FAST Exam Real Time Training Model (item #: BP-FAST1800), Blue PhantomTransparent Internal Jugular Central Line (item #: BPIJ500-C), Blue Phantom Regional Anesthesia and Central Line Model (item #: BPHNB670), Blue Phantom Midscapular thoracentesis model (item #: BPTT2-1005), and the CAE/VIMEDIX: Transthoracic ECHO simulator.

In February 2013, 208 students from eight medical and allied health schools attended Ultrafest (University of California, Irvine, Los Angeles, Davis and San Diego, University of Southern California, Loma Linda University, Touro University and Western University). All participating students were enrolled in MD (155, 75%), DO (38, 18%), or PA (15, 7%) programs. Twenty physicians from obstetrics and gynecology, anesthesia, emergency medicine, and internal medicine from UC Irvine, Davis and San Francisco, Stanford and Loma Linda served as workshop leaders. Twenty-four well-trained UC Irvine medical students served as small group instructors, with student to instructor ratio <5:1. All attendees participated in five one-hour workshops in addition to four hours of didactic online preparatory training, for nine total instructional hours.

Forty-eight students (38 MD, 8 DO, 2 PA) consented to this cohort study to evaluate change in practical knowledge and clinical skills in US before and after Ultrafest. Students were randomly assigned to be evaluated for proficiency in Pulmonary US or FAST, assessing clinical knowledge through written exam and image acquisition. We did not assess prior US training for the volunteer subjects. These volunteers were required to participate in the Pulmonary US or FAST course, which required some volunteers to change their preferences to include one of these courses. All students in the study were assigned to review the pre-course online didactic material for Pulmonary US and FAST.

The instruction at all stations, including the Pulmonary US and FAST stations was standardized to include specific information detailed in handouts given to each of the instructors prior to Ultrafest. This ensured that each instruction goal was met during their hands on course, and provided appropriate training to perform and interpret point of care ultrasound.

### Course Content

Each station included instruction on the ideal probe to use, optimal probe placement for image acquisition and the interpretation of relevant anatomy for each ultrasound study. The Pulmonary US station also included specific instruction on the identification and clinical significance of A lines and B lines, how to identify a pneumothorax using both “b” and “m” modes, the recognition of lung sliding and various signs including the “sky, ocean, beach” sign and the “barcode” sign, as well as how to identify pleural effusions and to recognize significant artifact including mirror imaging. While the live models did not have pulmonary pathology, the students were able to identify pathology of pleural effusion through phantom models and were provided images of pneumothorax examples. The FAST US focused on acquisition of the four windows of the FAST exam, including subxiphoid cardiac window, hepatorenal recess, splenorenal recess, and suprapubic views. The FAST exam also emphasized identifying anechoic free fluid as well as the recognition of clinically relevant artifacts including reverberation, mirror image artifact, posterior enhancement and edge artifact. While the live models did not have pathology in the FAST stations, phantom models with positive FAST scans in addition to image clip examples were provided for the students.

Pre-Ultrafest examinations were done during the hour prior to the conference, with post examinations immediately after. All testing was proctored. Students from the host university were excluded due to high baseline exposure to US and teaching materials. Students who completed the study were compensated with an US textbook. The study was approved by the local institutional review board. Primary outcome measure was improvement in multiple-choice questions focused on pulmonary or FAST US knowledge. Secondary outcome was improvement in image acquisition for four windows on standardized models in either pulmonary US or FAST.

### Clinical Knowledge

Clinical knowledge was assessed by 21 written multiple-choice questions for either Pulmonary US or FAST. Questions were written by the US director and focused on practical knowledge for diagnosis and image interpretation. Paired t-test analysis was done on total scores of the pre- and post-examinations for both the Pulmonary US and FAST groups.

### Clinical Skills

We assessed clinical skills by ability of students to acquire four windows each in pulmonary US and FAST. For FAST, participants were instructed to scan the right upper quadrant, left upper quadrant, subxiphoid and suprapubic windows. For pulmonary US, participants scanned windows to evaluate pleural effusion, A and B lines, pneumothorax in 2D and then M-mode. Proctors saved image clips when students expressed verbal satisfaction with image quality. Participants were not given feedback about quality of images or instructed how to improve. Images were saved with a coded label to identify and match students.

Images were scored by the US director, blinded to student identification and timing of assessment, as unacceptable, acceptable, or excellent. We calculated overall score for each exam as the sum of four window components of each exam, with 0 points (unacceptable), 2 points (acceptable), and 3 points (excellent) assigned. An image was “unacceptable” if it could not reveal potential pathology. Acceptable images visualized the organs of importance to identify pathological changes if present. An additional point was given for an excellent image with proper gain, depth, location and scanning technique. The scoring reflected a larger difference between an unacceptable [0] and acceptable [2] image, than between acceptable and excellent [3], to reflect greater clinical import of an optimum diagnostic image. We conducted paired t-tests on pre- and post-data, with students as their own controls.

## RESULTS

### Clinical Knowledge

**Figure 1:**
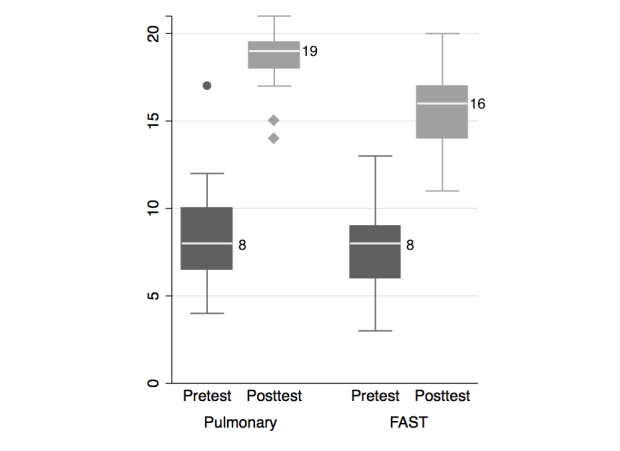
Boxplot of written Clinical Knowledge pre- and post-Ultrafest scores for Pulmonary Exam and FAST (Focused Assessment of Sonography for Trauma) n =22.

We analyzed data for 46/48 subjects (two excluded for incomplete written exams). Twenty-four (of 46) completed pre- and post-Ultrafest pulmonary US written exams. Pulmonary knowledge scores increased by mean 10.1 points (95% CI [8.9–11.3], p<0.00005), from a pretest average 8.4, to posttest average 18.5 of 21 possible points (Figure 1). Twenty-two students completed the FAST pre- and post-Ultrafest written exams. FAST knowledge scores increased by mean 7.5 points (95% CI [6.3–8.7] p<0.00005), from pretest average 8.1 to posttest average 15.6 of 21 possible points (Figure 1). For neither application were there statistically significant pre-post differences by medical student year of training.

### Clinical Skills

**Figure 2:**
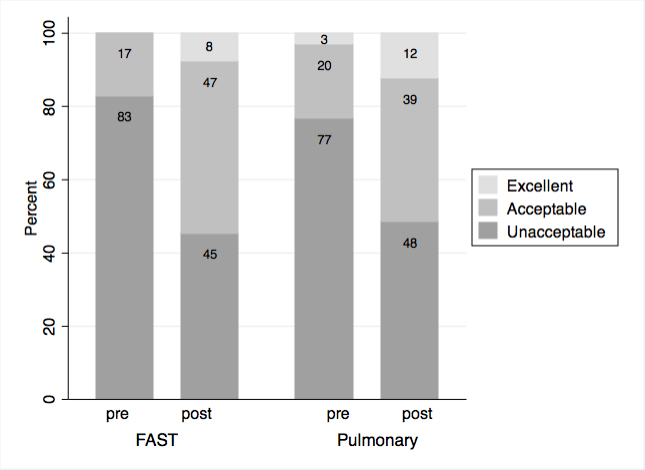
Percent improvement in Clinical Skills Image Acquisition between pre- and post-Ultrafest for FAST (Focused Assessment of Sonography for Trauma) and Pulmonary Ultrasound exams. N=16 for each ultrasound application.

We analyzed image acquisition data on 32/48 students (66%, 16 each in pulmonary US and FAST) by both paired t-test and overall percent improvement. Mean score improved from 1.7 pre- to 4.7 posttest (of 12 possible points). Therefore, the average posttest performance did not meet the eight points needed for adequate image acquisition in all four windows (2 points each). For both studies combined, mean improvement was 3.0/12 (95% CI [2.0–3.9], p<0.00005), FAST exam alone improved by 3.3 (95% CI [2.0–4.6], p=0.0001), and pulmonary exam improved by 2.6 (95% CI [1.1–4.2], p=0.003). The data show that image acquisition on both exams combined improved from 80% unacceptable, 19% acceptable, and 2% excellent, to 47% unacceptable, 43% acceptable, and 10% excellent. The views for the two modalities are separated in Figure 2.

## LIMITATIONS

These data are limited by small sample size and moderate drop-out rate for 16 inadequate image clips. The 48 volunteers recruited may have had additional interest, aptitude or experience with US, and could have inflated the measured improvement. We made no attempt to determine whether and to what extent students carried these skills to the clinical bedside after training, or whether they retained what they learned. Image quality assessments of “unacceptable,” “acceptable” and “excellent” were subjective and unvalidated. Student preceptors may have coached students contrary to instruction. We did not check or validate students’ viewing of pre-course materials.

We did not assess prior US training or expertise in our volunteer subjects. This would be a confounding variable in our assessment methods. Our study design did not discriminate between the value of the pre-course didactics, the one- hour lecture and the hands-on training.

## DISCUSSION

Research on the efficacy of ultrasound in both medical education and patient care is expanding. Results from multiple studies support using ultrasound to facilitate and supplement anatomy courses in medical education.^5^,^6^ Additional research suggests direct clinical benefit from bedside US, demonstrating improved physical exam skills and overall increased confidence in medical students using US.^7^ Furthermore, one study showed medical students who were taught US achieved greater accuracy collecting specific physical exam data when compared to experienced physicians not using US.^8^ Such studies suggest that physician-performed bedside US may enhance diagnostic accuracy and therefore lead to more informed treatment decisions. However, most medical schools in the United States do not have integrated US curricula, leaving most students without instructors or machines to learn on. While many medical schools may be interested in including such training, there are many limitations, including lack of funding and faculty resources to support such an endeavor.

This study sought to determine whether hands-on US training, outside traditional medical school curricula in a day-long symposium, could improve student knowledge and skills. The effectiveness of similar short (<6 hours) hands-on training models in medical education has been supported by other studies in a variety of fields including surgery and BLS.^9^,^10^ Student interest in US training symposia is demonstrated by robust attendance for two consecutive years. Subjective evaluations of the symposium have also been overwhelmingly positive.^4^

Our cohort study on Ultrafest suggests that a daylong symposium on US is effective in improving clinical knowledge but not in achieving adequate image acquisition for pulmonary and FAST ultrasound assessments. We found significant improvement in written clinical knowledge exam by almost every student, suggesting students achieved clear advances in understanding of the US examinations and interpretations. Although we found significant improvement in practical image acquisition, almost half of these were still judged inadequate after training. This implies that most students were poorly skilled at image acquisition prior to training, and that the one-hour subject-specific workshop was insufficient to achieve proficiency. Further studies are needed to assess clinical applicability of these US techniques, and those from the other workshops, in clinical practice.

The complexity of hands-on training in US for large groups presents many challenges, including assessing student improvement and proficiency, and evaluating integration of new skills with patient care. As each patient is unique in body habitus, pathology and cooperation, each US application requires integration of many user skills that are difficult to assess. In addition, skill in image acquisition does not reliably generalize across different US examinations, as each requires training on required windows.^11^ The amount of training required to become proficient in each examination also seems to differ.^12^

While free US symposia for hundreds of students may offer significant benefits, they require extensive preparation, experienced volunteers, and funding. Therefore, such symposia may be unsustainable. This symposium included 36 ultrasound machines, seven phantom models, finances for food and special pelvic and genitourinary models ($200–$400 each), 32 live volunteer models, 20 volunteer physician instructors, 24 trained US medical student instructor volunteers, and adequate space to hold workshops in 15 breakout rooms. However, the paucity of US training capability among medical schools may make central training at centers of excellence viable, with pooling of financial resources.

Although the hands-on component of training was the primary purpose of Ultrafest, we used a written examination with questions focused on the acquisition and interpretation of the corresponding ultrasound study as a primary outcome measure. Practical examinations in the two modalities were used as secondary endpoints, as this is a non-traditional way of assessing ultrasound skills. There is no practical examination validated at this time to evaluate a clinician’s ultrasound skills.

For example, the national American Registry of Diagnostic Medical Sonographers credential assessment is a multiple-choice format written examination, though it seeks to measure knowledge on performing and interpreting ultrasound images. We did not similarly grade US images for Ultrafest, as models had different anatomy, no pathology, and image grading is subjective. The written exam we used by contrast, is more objective and reflects pathology. We have attached the test questions as Appendix I. There has been a recent transition of teaching ultrasound at the bedside as opposed to in a lecture hall which has been shown to improve learning.^14^

Future symposia may foster greater proficiency in US skills by lengthening the hands-on workshops and expanding the symposium to two or more days. One small study supports a two-day model where PGY 1 residents, novice to ultrasound, participated in two, four-hour blocks over two days in physics, FAST, cardiac, aorta, renal, gallbladder, and pelvic sonography. Although the study was limited by sample size (n =12), they found significant improvement which persisted for six months without additional training.^13^

Future studies should evaluate students longitudinally for shor-t and long-term (3–6 months) retention of knowledge and psychomotor skills.

## CONCLUSION

Physician performed bedside US is a promising adjunct to traditional physical examination. Its major limitation is physician training, as most medical schools have not yet integrated US. A one-day, nine-hour, small group instruction and practice symposium improved student knowledge on trauma and pulmonary US, and improved image acquisition, but the latter fell short of significant proficiency. While many improvements can be made to this symposium, this model suggests that central training centers of excellence may be a viable option for US training in medical education.

## Supplementary Information
