# Supplementary material for: Ultrafest: A Novel Approach to Ultrasound in Medical Education Leads to Improvement in Written and Clinical Examinations
Source: West J Emerg Med. 2014 Dec 15;16(1):143–8. doi: 10.5811/westjem.2014.11.23746 (PMC4307699; doi:10.5811/westjem.2014.11.23746)

FAST:

1) In the subxiphoid view, where is the indicator pointed?
  a. Patients left
  b. Patients head
  c. Patients left hip
  d. Patients right

2) On a FAST exam, what do anechoic regions usually indicate?
  a. Artifact
  b. Free Fluid
  c. Posterior Enhancement
  d. Reverberation

3) Which image is not included as part of the FAST exam ?
  a. Subxiphoid
  b. Bladder
  c. Splenorenal Recess
  d. Abdominal Aorta

4) Which probe is most often used for the FAST exam?
  a. P21
  b. Endocavitary
  c. L18
  d. L38

5) At which anatomical site can you find Morison's Pouch?
  a. Right Midaxillary Line
  b. Suprapubic
  c. Left Midaxillary Line
  d. Subxiphoid

6) What artifact is lost when fluid accumulates in the right lung?
  a. Reverberation
  b. Mirror image artifact
  c. Posterior Enhancement
  d. Edge Artifact

7) Which cardiac window is often used if the subxiphoid view does not reveal an adequate image?
  a. Parasternal Long
  b. Apical 4 chamber
  c. Suprasternal
  d. Apical 5 chamber

8) Which window usually requires the most posterior placement of the probe?
  a. Subxiphoid
  b. Morrison's Pouch
  c. Splenorenal recess
  d. Suprapubic

9) What does free fluid look like on an Ultrasound Exam?
  a. Anechoic
  b. Hyperechoic
  c. Heterogenous
  d. White

10) Compared to the spleen, the liver should be roughly:
  a. Isoechoic
  b. Anechoic

c. Hyperechoic
d. Hypoechoic

11) Compared to most surrounding structures, the bladder is:
a. Hyperechoic
b. Isoechoic
c. Heterogenous
d. Hypoechoic

12) What finding may be misinterpreted as intraperitoneal free fluid?
a. Perinephric Fat Pad
b. Renal Cysts
c. Hydronephrosis
d. Dilated IVC

13) What do you use as an acoustic window to view the heart in the subxiphoid view?
a. IVC
b. Kidney
c. Lung
d. Liver

14) How does the diaphragm appear compared to most surrounding structures?
a. Hypoechoic
b. Heterogenous
c. Loculated
d. Hyperechoic

15) What window of the heart is shown below?
a. Parasternal Long
b. Subxiphoid
c. Apical 4
d. Apical 5

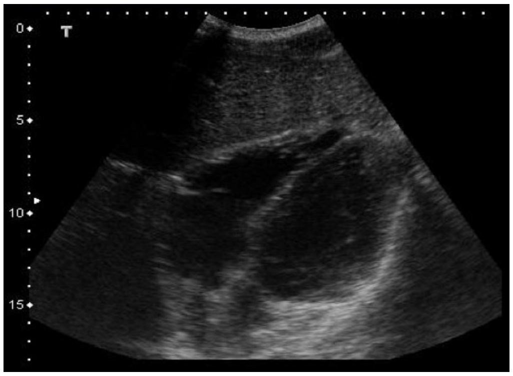

16) What potential space exists in the image below?
   a. Morison’s Pouch
   b. Pouch of Douglas
   c. Splenorenal recess
   d. Vessicouterine Junction Pouch

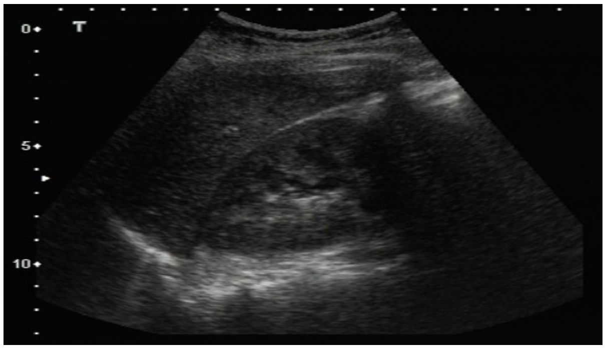


17) What does the image below show?
   a. Perinephric Fat Pad
   b. Hydronephrosis
   c. Pleural Effusion
   d. Positive FAST Scan

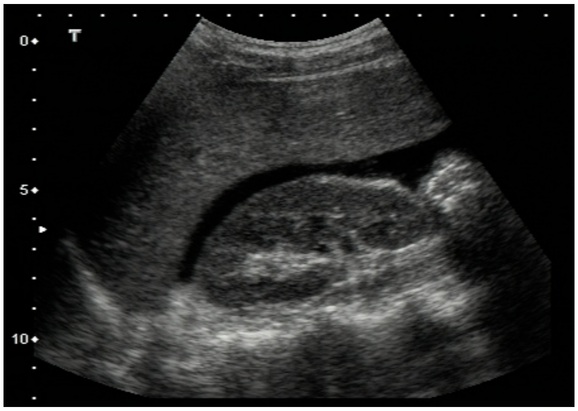


18) What does the suprapubic image below show?
   a. Fluid in the Pouch of Douglas
   b. Normal image
   c. Fluid in the Vessicouterine Pouch
   d. Ovarian Cyst

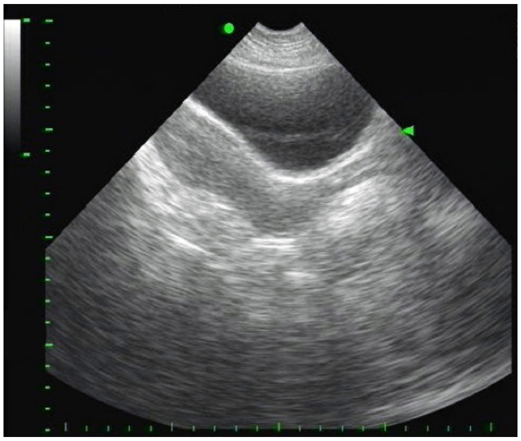

19) What pathology does the image below show?
   a. Pleural Effusion
   b. Intraperitoneal fluid
   c. Ascites
   d. Normal Scan

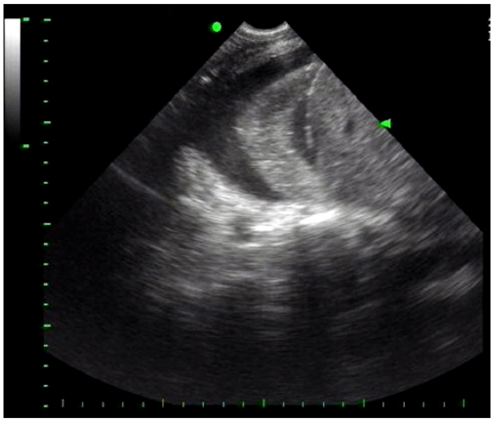

20) What is the most hyperechoic structure on the image below?
   a. Liver
   b. Lung
   c. Diaphragm
   d. Duodenum

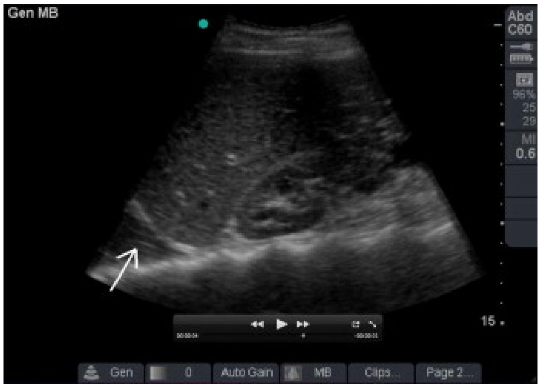


21) What artifact is shown in the image below?
   a. Reverberation
   b. Mirror image artifact
   c. Posterior Enhancement
   d. Edge Artifact

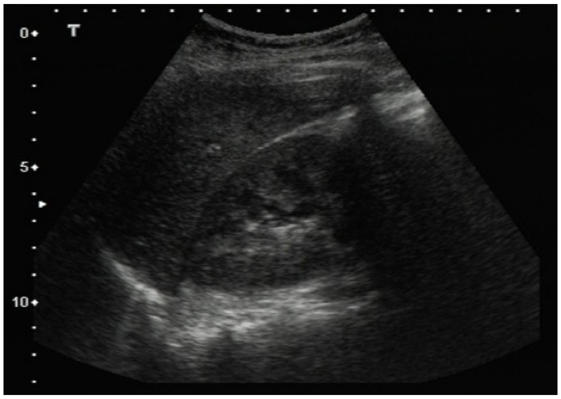

<u>PULMONARY:</u>

1) Where is a pneumothorax best identified when the patient is supine?
   a) posterior/lateral zone
   b) all zones
   c) anterior zone
   d) apical zones

2) What are B lines?
   a) horizontal artifact
   b) pleural line
   c) rib shadows
   d) vertical artifact

3) What are A lines?
   a) horizontal artifact
   b) pleural line
   c) rib shadows
   d) vertical artifact

4) What do B lines indicate?
   a) Fluid consolidation
   b) Air
   c) Pneumothorax
   d) Pleural Effusion

5) What do A lines indicate?
   a) Fluid consolidation
   b) Air
   c) Pneumothorax
   d) Pleural Effusion

6) What setting do you use to visualize the sky-ocean-beach sign?
   a) Color
   b) Doppler
   c) 2D
   d) M Mode

7) A patient with primarily COPD is more likely to have what predominant pulmonary images?
   a) A-lines
   b) B-lines
   c) “bar code” sign
   d) lost mirror image artifact

8) A patient with primarily CHF is more likely to have what predominant pulmonary images?
   a) A-lines
   b) B-lines
   c) bar code” sign
   d) lost mirror image artifact

9) Which of the following is an indicator that the lung pleura is in tact in 2D mode?
   a) A-lines
   b) B-lines
   c) “barcode” sign
   d) “ants marching on a string” sign

10) Which of the following indicates a pneumothorax in M-Mode?
   a) A-lines
   b) B-lines
   c) “barcode” sign
   d) “ants marching on a string” sign

11) <u>The loss of</u> which of the following indicates a pneumothorax in 2D mode?
   a) A-lines
   b) B-lines
   c) “barcode” sign
   d) “ants marching on a string” sign

12) What does an image of the parietal and visceral pleura sliding look like in 2D?

a) A-lines
b) B-lines
c) “barcode” sign
d) “ants marching on a string” sign

13) Where is a pleural effusion best identified?
a) Posterior/lateral zone
b) all zones
c) anterior zone
d) apical zones

14) What is it called when both the collapsed lung (pneumothorax) and intact lung are captured in the same image?
a. Mickey Mouse Sign
b. Playboy Bunny Sign
c. Sky-Ocean-Beach
d. Lung Point

15) What artifact indicates that there is NOT a pleural effusion?
a. Mirror Image
b. Reverberation
c. Posterior Enhancement
d. Acoustic Shadowing

16) What pathology does the image below show?
a. Pleural Effusion
b. Intraperitoneal fluid
c. Ascites
d. Normal Scan

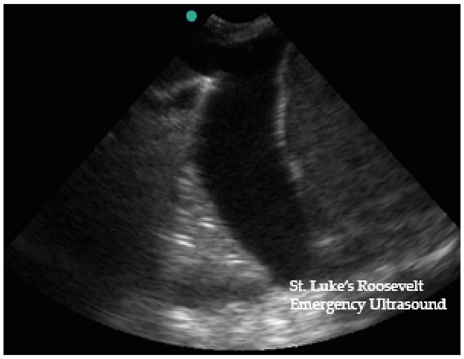


17) What is shown in the image below?
a) A-lines
b) B-lines
c) “sky-ocean-beach” sign
d) “barcode” sign

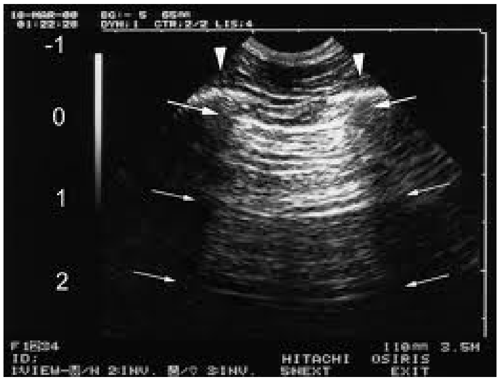

18) What is shown in the image below?
a) A-lines
b) B-lines
c) “sky-ocean-beach” sign
d) “barcode” sign

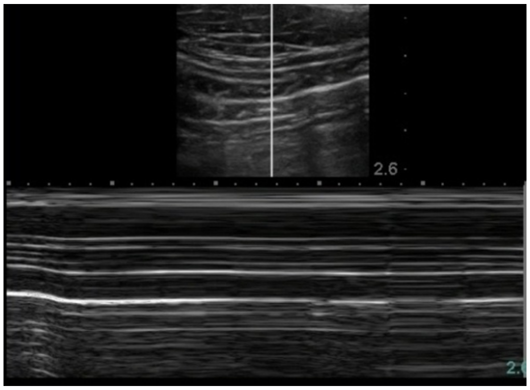


19) What is shown in the image below?
a) A-lines
b) B-lines
c) “sky-ocean-beach” sign
d) “barcode” sign

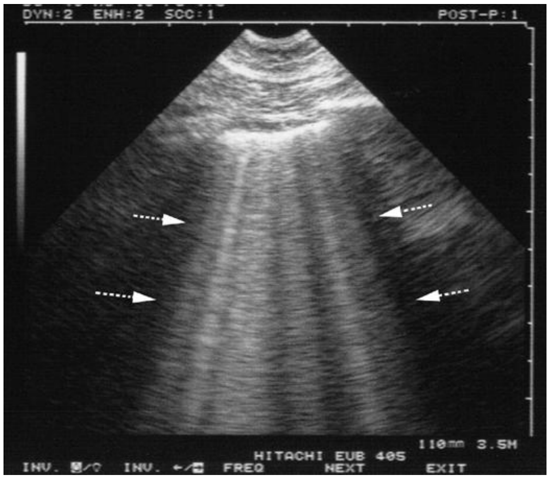


20) What is shown in the image below?
a) A-lines
b) B-lines
c) “sky-ocean-beach” sign
d) “barcode” sign

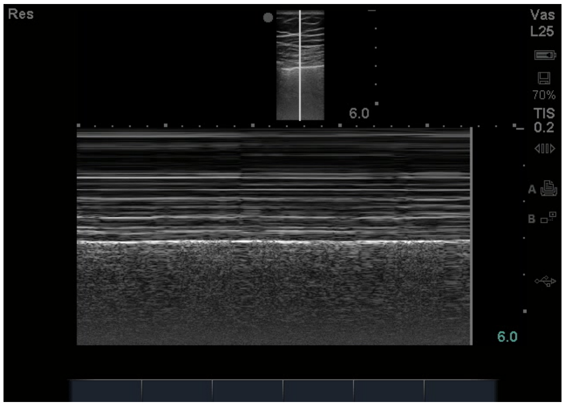

21.) What does the image below indicate?

a. Normal Lung
b. Pneumothorax
c. COPD
d. CHF

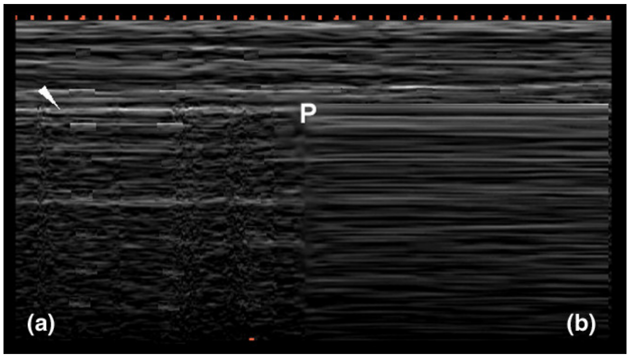


22.) What artifact is evident in the image below?

a. Mirror Image
b. Reverberation
c. Posterior Enhancement
d. Acoustic Shadowing

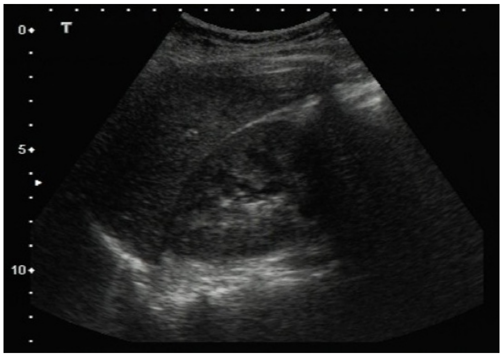

Supplement: Supplementary file 1 [file wjem-16-143-s001.pdf]
